# Pilot-testing an adverse drug event reporting form prior to its implementation in an electronic health record

**DOI:** 10.1186/s40064-016-3382-z

**Published:** 2016-10-11

**Authors:** Adam Chruscicki, Katherin Badke, David Peddie, Serena Small, Ellen Balka, Corinne M. Hohl

**Affiliations:** 1 Faculty of Medicine, Queen’s University, 15 Arch Street, Kingston, ON K7L 3N8 Canada; 2 Department of Emergency Medicine, University of British Columbia, 855 West 12th Avenue, Vancouver, BC V5Z 1M9 Canada; 3 School of Communication, Simon Fraser University, 8888 University Drive, Burnaby, BC V5A 1A6 Canada; 4 Centre for Clinical Epidemiology and Evaluation, Vancouver Coastal Health Research Institute, 828 West 10th Ave, Vancouver, BC V5Z 1M9 Canada

**Keywords:** Adverse drug events, Pilot-testing, Electronic medical records, Reporting

## Abstract

**Background:**

Adverse drug events (ADEs), harmful unintended consequences of medication use, are a leading cause of hospital admissions, yet are rarely documented in a structured format between care providers. We describe pilot-testing structured ADE documentation fields prior to integration into an electronic medical record (EMR).

**Methods:**

We completed a qualitative study at two Canadian hospitals. Using data derived from a systematic review of the literature, we developed screen mock-ups for an ADE reporting platform, iteratively revised in participatory workshops with diverse end-user groups. We designed a paper-based form reflecting the data elements contained in the mock-ups. We distributed them to a convenience sample of clinical pharmacists, and completed ethnographic workplace observations while the forms were used. We reviewed completed forms, collected feedback from pharmacists using semi-structured interviews, and coded the data in NVivo for themes related to the ADE form.

**Results:**

We completed 25 h of clinical observations, and 24 ADEs were documented. Pharmacists perceived the form as simple and clear, with sufficient detail to capture ADEs. They identified fields for omission, and others requiring more detail. Pharmacists encountered barriers to documenting ADEs including uncertainty about what constituted a reportable ADE, inability to complete patient follow-up, the need for inter-professional communication to rule out alternative diagnoses, and concern about creating a permanent record.

**Conclusion:**

Paper-based pilot-testing allowed planning for important modifications in an ADE documentation form prior to implementation in an EMR. While paper-based piloting is rarely reported prior to EMR implementations, it can inform design and enhance functionality. Piloting with other groups of care providers and in different healthcare settings will likely lead to further revisions prior to broader implementations.

## Background

Adverse drug events (ADEs) are harmful and unintended consequences of medications that account for 1.8 million emergency department visits in Canada each year, and are a leading cause of unplanned admissions (Hohl et al. 2001; Zed et al. 2008; Budnitz et al. 2011). Despite the significant burden that ADEs pose on patients and the healthcare system, they are often not documented by clinicians, nor effectively communicated between health professionals or across healthcare settings (Hohl et al. 2005, 2010), contributing to unintentional re-exposures of culprit drugs and repeat ADEs (Zhang et al. 2007). Van der Linden et al. (2006) estimate that 27 % of medications withdrawn during hospitalization due to an ADE are re-prescribed within only 6 months, indicating an urgent need to develop electronic systems that can help clinical care providers prevent repeat unintentional exposures to harmful drugs. In a recent systematic review, underreporting of ADEs by healthcare providers was identified as the main reasons why the effectiveness of current electronic systems to prevent unintentional re-exposures is limited (Van der Linden et al. 2013). Improved, structured electronic documentation of ADEs may facilitate the creation of patient-specific report that can be used to generate medication-level or medication class-level alerts to prevent unintentional re-exposures (Van der Linden et al. 2015).

While many electronic medical records (EMRs) provide dedicated space allowing care providers to record ADEs, most are focused on allergy information and do not provide the option for structured reporting (Van der Linden et al. 2013). A systematic review of ADE reporting systems that are external to EMRs (e.g., Health Canada’s Medwatch program) found wide variation in the variety and type of ADE data collected (submitted). None of the systems reported pilot-testing electronic fields prior to their implementation to ensure user-friendliness, succinctness, relevance and correct interpretation of fields by care providers. In participatory workshops completed by our group reflecting the views of over 120 care providers in varied clinical settings, the length of ADE reporting forms, the time required to complete them, duplication of information requested, and lack of relevance to clinical care were barriers to ADE documentation (submitted and unpublished data). Creating structured, succinct and clear ADE data input fields in electronic record systems that can be leveraged to create patient-specific safety alerts to avoid unintentional re-prescribing was perceived as an incentive to report.

The design of electronic information systems in healthcare is complex, as systems must be user-friendly, meet the needs of multiple end-user groups, require approval from a broad range of stakeholders, and be adaptable to multiple environments (Kushniruk 2002). New systems implemented without pilot-testing and refining often fall short of anticipated goals due to design failures, or systems’ architecture constraints that could have been identified and addressed prior to their final build. This is particularly evident in healthcare where the rapidly changing user needs, incomplete information and shifting goals can derail even the most meticulously planned system implementation (Kushniruk 2002). Pilot studies provide an opportunity to evaluate new concepts at intermediate stages of design (Tejilingen et al. 2001). However, piloting electronic data forms is time-consuming and costly, as reprogramming is required to introduce refinements. Instead, pilot-testing paper-based forms may yield more advanced designs at lower cost and save the time and costs required to reprogram interfaces (Grady 2000). Paper-based, iterative design has been widely adopted by software developers, yet few examples of this approach in healthcare systems design exist (Anderson et al. 2001; Girsedale et al. 1997). Our objective is to describe paper-based piloting of a new electronic ADE documentation platform that aims to enhance communication between providers, and the design insights that resulted from this process.

## Methods

### Design and setting

This qualitative study was part of a larger research program, which has as its goal the design and implementation of an electronic ADE reporting system within an EMR at thirteen hospitals (Peddie et al. 2016). This study was conducted in the emergency departments and hospital wards of Vancouver General Hospital (VGH), a tertiary care referral, and Lions Gate Hospital (LGH), an urban community hospital in British Columbia, Canada, between June and August, 2015.

### Compliance with ethical standards

The University of British Columbia Clinical Research Ethics Board approved the study protocol (H13-02316-009). We obtained verbal informed consent from all participants. This study was funded by the Canadian Institutes of Health Research (Grant No. 2935460). None of the authors have any conflicts of interest to declare.

### Study participants

We enrolled a convenience sample of key informants, all of whom were clinical pharmacists working in settings with a high ADE prevalence. We recruited participants by sending email invitations to all clinical pharmacists working at both institutions through the Department of Pharmaceutical Sciences, and encouraged volunteers to recruit colleagues by word-of-mouth. The only inclusion criterion was that participants actively practice clinical pharmacy at a participating hospital.

### Design of the paper-based ADE form

**Fig. 1:**
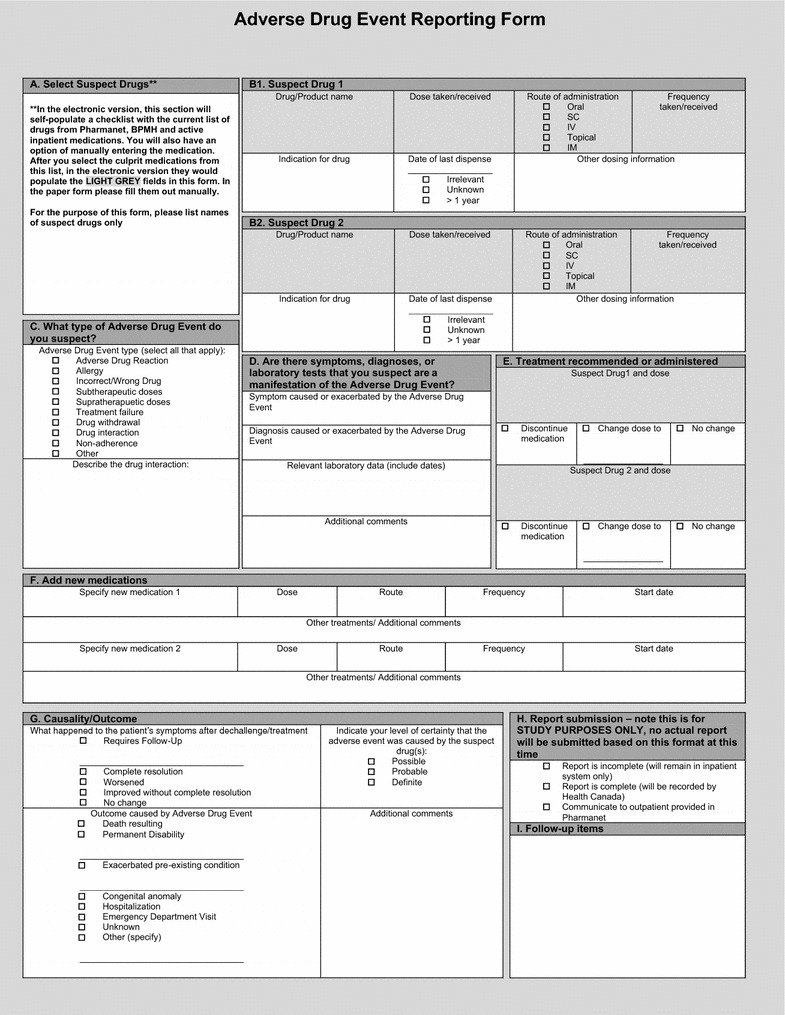
Paper-version of the electronic ADE reporting form

We previously identified a minimum required dataset for ADE reporting by conducting a systematic review of the literature (submitted), and eight participatory workshops with over 120 physician and pharmacist end users working in inpatient and outpatient settings across our healthcare region. The participants identified which data fields were relevant, and proposed a sequence in which fields should be presented to end-users to enhance functionality. We used this information to draft a paper-based version of the electronic reporting form (Fig. 1), and organized individual ADE fields into boxes labeled A to I to help with the progression of the form (Table 1). The electronic version will use auto-populating fields that contain medication dispensing information from drug plan data which we could not represent in the paper-based form.

**Table 1:** Data collection fields used in the paper version of the ADE reporting form

Concept	Data field	Field description
Name and dose of the culprit drug(s)	A. Select suspect drug(s)	Enter a list of suspected drugs
	B. Suspect drug(s)	Enter the name(s), dose, route of administration, frequency, indication for, date of last dispense, and other relevant information about the suspect drug
Effect(s) of the ADE on the patient	C. What type of ADE do you suspect?	Select the type of ADE that occurred, if it was a drug-drug interaction, describe it in more detail
	D. Are there symptoms, diagnoses, or laboratory tests that you suspect are an ADE manifestation?	Describe the symptoms and/or diagnosis caused or exacerbated by the ADE. Include relevant laboratory data and additional comments
Treatment received	E. Treatment recommended or administered	Describe the treatment for the ADE
	F. Add new medications	List any newly recommended medications
Outcome	G. Causality and outcome	Describe what happened to the patient’s symptoms after treatment, the outcome of the ADE, and indicate the level of certainty that the event was caused by the suspect drug. List additional comments
	H. Report submission	Indicate whether the report should remain in the inpatient record, be submitted to Health Canada or communicated to the drug plan
	I. Follow-up items	List additional comments

### Data collection

We used lightweight ethnography, a social science approach allowing investigators to collect specific and relevant information efficiently while accepting the impossibility of a complete understanding of a work setting (Randall et al. 2007). Lightweight ethnography can provide guidelines for technology design as it is neither time nor resource intensive, and is well suited for pilot studies. One research assistant (AC) shadowed clinical pharmacists during 2–4 h data collection shifts. At the beginning of each observation shift, the research assistant presented pharmacists with the ADE form and explained the form functionality, as well as the scope of ADEs that it is aimed to capture. Pharmacists were asked to complete the form when an ADE was encountered. In addition to collecting ethnographic information, the research assistant recorded how long it took users to complete the form, from the moment they started filling it out to when it was ready to be submitted. The research assistant also recorded the types of information sources accessed and the number of internal and external interruptions to pharmacists’ work while completing the form. Internal interruptions were defined as instances where the user had to access another information source in order to complete the form. External interruptions were defined as instances where another person interrupted the user completing the ADE form. The research assistant recorded pharmacists’ comments and impressions during work, and subsequently completed semi-structured interviews with pharmacists about the diagnostic process, users’ perceptions of individual fields and the form as a whole, and challenges in documenting ADEs. We collected all completed ADE reporting forms after each shift.

### Data analysis

We calculated the proportion of completed data fields for individual ADE reporting forms by taking the number of data fields completed by the user and dividing this number by the twenty-seven data collection fields contained on the ADE form. An individual data collection field was defined as one unique question-response pair. A data field was marked as filled, if the user provided either a written response or checked off a tick-box. The form was divided into boxes labeled A to I. Boxes B, “Suspect drug”, and F, “Add new medication”, contain duplicate spaces to accommodate users entering more than one drug at a time. If only one culprit drug was suspected, the duplicate space was left out of the average data field completion rate calculations. If two culprit drugs were suspected, the duplicate space was included in calculations. To calculate the average data field completion rates across all users, we averaged the individual completion rates from all the collected forms. The average and standard deviation for the number of interruptions and time to complete the form were used to calculate the 95 % confidence intervals. We transcribed field notes and coded data using NVivo 10, a qualitative data analysis software that can be used to interrogate and analyze unstructured qualitative data. We combined inductive reasoning (moving from particular to general) and constant comparison (generalizing concepts and categories) to code the data for themes related to the ADE form, challenges in diagnosing and reporting ADEs, and workflow.

## Results

### ADE reporting

We observed six clinical pharmacists for 25 h across 11 data collection shifts at VGH and LGH. During this time, pharmacists completed 24 ADE forms. The clinical pharmacists perceived the paper-based ADE form as an efficient, user-friendly, and intuitive way to record ADEs. Users preferred the form on a single page, and preferred checkboxes over drop-down menus and free text. They also felt that some data entry fields could be omitted (Table 2). For example, pharmacists indicated that section B, “Suspect Drug”, should be better at capturing the order in which medications were prescribed. Pharmacists also felt that subsection of “Suspect Drug”—“date of last dispense” lacked utility and could be removed. Users felt that instructions for some fields needed further clarification or simplification. For example, section F, “Add new medications”, required clarification. Pharmacists were unsure about whether to list medications used to treat an ADE, a drug that was prescribed to replace a culprit medication that was discontinued, or both. They also thought that section H, “Report submission” was confusing, as it contained multiple reporting options (Table 2).

**Table 2:** Comments and proposed resolutions

Data field	Comments	Proposed solution
A. Select suspect drug(s)	None	NA
B. Suspect drug(s)	“Date of last dispense” is irrelevant Difficult to capture order of prescribing Difficult to enter complex dosage regimes	Remove the “date of last dispense” field Increase the amount of free-text entry Remove one data entry box for drugs (to autopopulate in electronic form)
C. What type of ADE do you suspect?	Checkboxes preferred over drop-down menus Provide the option to describe “other”	Use check boxes instead of drop-down menus Modify the free-text option
D. Are there symptoms, or laboratory tests that you suspect are an ADE manifestation?	Provide space to list vital signs	Add option to add vitals in the “laboratory data” section
E. Treatment recommended or administered	Need to be able to input start and stop time of the changes	Add a “start” and “stop” date data input
F. Add new medications	Name of field is confusing Unsure about which medications to list	Change the name of the box to clarify the instructions
G. Causality and outcome	Pharmacists often don’t know the patient’s outcome Pharmacists would like to pass the form to another care provider for completion (e.g., GP)	Provide option for other care provider(s) to complete symptom resolution and outcome reporting. Requires linkage to MD electronic data entry
H. Report submission	Improve clarity of instructions for inpatient reporting Pharmacists are hesitant to report without a definite diagnosis, especially if their identification is attached to the report	Simplify reporting options Add option to remove or modify existing report(s) Educate pharmacists that the form is primarily to improve documentation and communication between care providers, rather than to report Change name of form to “documentation and communication” to clarify intent
I. Follow-up items	Field is unnecessary	Remove free-text boxes

*NA* not applicable, *MD* physician

During piloting, we observed that pharmacists interpreted the word “reporting” in our form to imply that its purpose was to generate an ADE report for Health Canada, something they felt was outside of the scope of clinical care provision.

### Barriers to reporting

Pharmacists were most likely to report more severe or rare ADEs, and adverse drug reactions were seen as the most “reportable” events. Pharmacists were generally hesitant to report ADEs when they were concerned about creating a permanent record without the ability to update, modify or delete it (e.g., if an alternative diagnosis became obvious at a later point in time), even though we emphasized that this would be possible in an electronic version. Pharmacists also thought that some ADE diagnosis might become irrelevant with time (e.g., a person with orthostatic hypotension caused by a high drug dose, who becomes more hypertensive with time and requires higher doses of said hypertensive medication). This indicated the need to for pharmacists to be able to re-access and modify electronic reports. Although pharmacists were familiar with the scope of ADEs intended to be captured by this form, some ADEs were challenging to recognize and diagnose. In these instances pharmacists remained uncertain about which events warranted documentation, indicating the need for education and guidance around this during the implementation phase of the electronic fields. They were also hesitant to report events in which they had not witnessed the patient’s outcome, as the outcome often impacted their causality assessment. Finally, they were reluctant to report suspected events. Most pharmacists initiated ADE documentation independently, without discussing the case with other care providers, even though an ADE diagnosis requires ruling out alternative diagnoses by the treating physician (e.g., ruling out of urinary tract infection prior to ascribing a diagnosis of delirium to a drug). Of completed forms, 15 of 22 (68 %) listed as outcome “requires follow-up,” highlighting the importance of enabling communication by allowing multiple care providers to access an electronic ADE report in both the inpatient and outpatient settings. This could enable communication whenever follow-up is provided in a different healthcare setting (e.g., after hospital discharge). Pharmacists were concerned that incorrectly reported suspect ADEs could conceivably hinder a patient’s future access to indicated medications. Thus, in an electronic design, reporters must be provided with the option of communicating with other care providers about new ADEs, as well as any changes to the patient status, as means of overcoming concerns about withholding what might be appropriate medications.

The average data field completion across all ADE forms was 50 % (95 % CI 47–53 %, n = 24). The least completed sections were D “are there symptoms, diagnoses, or laboratory tests that you suspect are an ADE manifestation?” of which, on average, only 41 % (95 % CI 35–47 %, n = 24) were complete. The low completion rate within section D was primarily due to the pharmacist deciding that only one field was necessary to express the problem caused by the ADE—generally either “symptom” or “diagnosis” (e.g. a cough caused by Ramipril did not require diagnosis and lab data, “GI bleed” was sufficient to describe an ADE to Rivaroxaban). Section F “add new medication” was only completed 19 % (95 % CI 6–32 %) of the time. Five out of six pharmacists generally completed one of four data collection fields in section D (symptoms, diagnosis, lab data, or additional comments), as not all fields were relevant to each ADE (e.g., lab values are only relevant when ADE has biochemically measurable outcomes). Pharmacists would usually fill out either the “symptom” or “diagnosis” of the ADE, but usually not both as this information was perceived as somewhat redundant. Our findings highlight the fact that form certain ADEs the report can be complete, even though users may not fill out all the data fields.

The most commonly reported ADEs were categorized as adverse drug reactions (16/24; 67 %) followed by drug–drug interactions (3/24; 13 %) and allergies (3/24; 13 %). The most common choice of treatment was discontinuation of the drug (15/24; 63 %). Pharmacists were reluctant to report any information about symptom resolution, and most commonly selected: “patient requires follow-up” (15/22; 68 %), as they were often not privy to this information at the time of reporting because symptom resolution would often occur only after discharge necessitating that another healthcare provider follow-up and complete the form. Follow-up was deemed necessary to report on alternative diagnoses that could rule out an ADE, and to update incomplete information (e.g., lab results).

The average time required to complete one form was 5.0 min (95 % CI 4.4–5.6 min, n = 12). The average number of internal interruptions was between 2 and 7, with a median number of 3 interruptions per form. Most interruptions occurred in order to access the printed patient’s outpatient medication dispensing record. Once implemented electronically, this step will be facilitated by prepopulating the ADE reporting form with a list of the patient’s dispensed medications, allowing pharmacists to tick the suspect drug(s) for the ADE.

## Discussion

Our objective was to pilot-test a paper-based version of a newly designed ADE reporting form in three clinical settings prior to integrating it into an EMR. Our work highlights the utility of pilot-testing health technology interventions by intended end-users within clinical settings in order to maximize user-friendliness, utility and relevance, even in situations in which end-users were involved in earlier design stages. While there are differences between electronic and paper data collection forms, the two approaches can produce synonymous results (Boyer et al. 2002; Huang 2006). Although not all functionalities of an electronic form can be mimicked by a paper-based form, crucial design elements required for a successful electronic implementation became apparent to end-users in paper-based testing and will influence our future electronic build. Our fieldwork helped end-users and researchers anticipate how the ADE form’s functionality could be improved to assist clinicians in communicating relevant ADE information between care providers on different wards and across healthcare sectors, and as handover tools. This enabled us to anticipate the need for electronic linkages between different components of the EMR being implemented, ideally including a bidirectional link with drug plan data.

One of our concerns at the outset was that the form would be too lengthy and require too much time to complete, distracting its users from other work duties. Surprisingly, our fieldwork did not confirm this, as most users completed the form within 5 min and generally approved of its length and level of detail. Although the paper-based version did not allow us to display future functionalities (e.g., pop-up windows, ability to revise ADE reports in the future), end-users were able to identify preferences when different design options were proposed. An important caveat is that additional features added in an electronic build may contribute to increased functionality, but may also add complexity and require more time, necessitating further refinements.

ADEs are vastly underreported using current ADE reporting systems (Hohl et al. 2013; Wiktorowicz et al. 2010). Our fieldwork identified important avenues for improving reporting that may be addressed in a future electronic ADE documentation and communication form that is integrated into an EMR. These include addressing uncertainty about which ADE types should be documented (possibly through pop-up instructions), allowing providers to document uncertainty in the ADE diagnosis, enabling reports to be removed or modified after follow-up, providing space for alternative diagnoses, and enabling inter-professional communication across handovers and between inpatient and outpatient settings (possibly via patient-specific safety alerts). In our study, the majority of reported ADEs were adverse drug reactions. Other kinds of ADEs, such as non-adherence, sub- or supra-therapeutic doses were seen as more complicated, as the implications of reporting were less clear. We used an extended definition of an ADE, which included non-compliance and improper dosing regiments. While, all these events fall under the scope of medication-related problems (MRPs), our form purposely avoided this term to increase signal to noise ratio, and prevent reporting multiple non-clinically significant events per patient, as our overarching goal was to prevent recurrence of serious ADEs while avoiding alert fatigue and rendering documentation feasible. In previous workshops we held with end-users in advance of pilot-testing, pharmacists’ insisted on retaining the option to record non-adverse drug reaction ADEs (unpublished data). This conundrum might be addressed by educating users about the various kinds of ADEs encountered and need for communication across providers, and supporting a common approach to preventing future ADEs.

This study confirms previous observational work by our group that suggests that ADE diagnosis is a complex and multi-step process (unpublished data). If ADE reporting is to succeed, electronic forms that are created for this process must reflect this complexity, and enable reporting as a multi-step process. Multiple care providers including those who provide insight into alternative diagnoses for suspect events or provide follow-up of patient outcomes must be able to access and update information. The immediate implication for the design of electronic reporting systems is that they must enable communication between providers and across healthcare sectors. While we piloted the form with clinical pharmacists, doctors and nurses in hospital and community settings are likely to utilize the form as well. Thus, we anticipate further piloting and design adjustments as the form is implemented in other healthcare environments and for other provider groups.

During our fieldwork, we referred to the ADE form as a reporting tool. However “reporting” was very specifically associated with the communication of a subset of events to Health Canada through MedEffect form, as opposed to their documentation within an electronic record. An implication of “reporting” was an assumed permanency of the record that would be created, as none of the currently available reporting mechanisms allow for updates or modification after a report has been generated. As the overarching objective of our project is to develop a documentation tool that supports communication between care providers (rather than communicate events to external agencies), we changed the name of our form to “Adverse Drug Event Communication and Documentation Form” to highlight its intended purpose. We hope that our findings highlight the need for a culture shift around ADE communication, from an approach that serves to generate health data for external agencies, implied by “reporting”, to a patient-safety oriented approach that focuses on communication and documentation for prevention of repeat events.

Low completion rates can indicate problems with availability of information needed to complete a section of the form, or content problems with the section itself. Among the sections with the highest non-completion rates were those for the ADE symptom and diagnosis. ADEs are notoriously difficult to diagnose, and our prior observational work and workshops with stakeholders suggested that providing a record which allowed a subsequent care provider to re-trace evidence upon which an ADE diagnosis was based as an important aspect of ADE documentation. Pharmacists often listed presumed ADE symptoms; however clustering them into a diagnosis can be challenging, or require communication with physicians to rule out alternative diagnoses or await the results of confirmatory testing, leading them to skip this field. This finding may also in part explain some of the uncertainty expressed by pharmacists about reporting more complex, or less traditional ADEs.

Pharmacists were often unclear about which treatment recommendations to list within the ADE documentation form (e.g., whether to document the medication used to treat the ADE, or a medication used to replace the culprit drug). As a result users would often leave this field empty. We were unable to capture the full functionality of our electronic form—which will enable the pharmacist to recommend changes to a patient’s medication regiment using the EMR to a physician who can approve of them or alter them. While the electronic build may contain sufficient contextual information to address the ambiguities which existed in the paper based version of the form, this is likely to require electronic piloting.

Our findings demonstrate the value of completing pilot studies of electronic health information technology implementations with paper-based forms. While these cannot mimic the full functionality of an electronic interface, they provided vital feedback for subsequent design and pre-implementation user education that we might have otherwise overlooked, including questions regarding systems architecture.

### Limitations

Lightweight ethnography, although time and resource efficient, carries a risk of only skimming the surface and providing only partial explanations. By only briefly engaging in the work environment, the observers risk missing less common routines and events that could otherwise have been captured. We relied on volunteers as the subjects of our observations, and therefore used a limited number of participants. This study is limited by the sole inclusion of clinical pharmacists, who were identified in our healthcare settings as the most likely care providers to encounter and document ADEs. It is possible that we might have uncovered other aspects of the ADE form requiring modifications had we been able to recruit more participants from other clinical backgrounds or settings, and anticipate further design adjustments as the use of the form is expanded. Also, our findings are susceptible to the Hawthorne effect, which occurs when participants are aware of the study objectives, potentially influencing their behavior. Finally, paper-based field evaluation of software designs has limitations, and hence findings must be evaluated in relation to data collected through other means.

## Conclusion

We piloted a paper-based version of an ADE documentation form prior to its electronic build. As a result, we were able to modify its design, and envision unique requirements for the system’s architecture as well as educational needs prior to system implementation. As a result of our pilot study, we will be able to address these issues to enhance functionality prior to an electronic build.

## Abbreviations

ADE: adverse drug event
EMR: electronic medical record
VGH: Vancouver General Hospital
LGH: Lion’s Gate Hospital
